# Investigating the Heavy Metal Adsorption of Mesoporous Silica Materials Prepared by Microwave Synthesis

**DOI:** 10.1186/s11671-017-2070-4

**Published:** 2017-05-04

**Authors:** Wenjie Zhu, Jingxuan Wang, Di Wu, Xitong Li, Yongming Luo, Caiyun Han, Wenhui Ma, Sufang He

**Affiliations:** 1 Faculty of Environmental Science and Engineering, Kunming University of Science and Technology, Kunming, 650500 China; 2 Faculty of Metallurgical and Energy Engineering, Kunming University of Science and Technology, Kunming, 650093 China; 3 State Key Laboratory of Complex Nonferrous Metal Resources Cleaning Utilization in Yunnan Province/The National Engineering Laboratory for Vacuum Metallurgy, Kunming University of Science and Technology, Kunming, 650093 China; 4 Key Laboratory of Non-Ferrous Metals Vacuum Metallurgy of Yunnan Province/Engineering Research Center for Silicon Metallurgy and Silicon Materials of Yunnan Provincial Universities, Kunming, 650093 China; 5 Research Center for Analysis and Measurement, Kunming University of Science and Technology, Kunming, 650093 China

**Keywords:** Microwave synthesis, Silica fume, MCM-41, Heavy metal adsorption

## Abstract

Mesoporous silica materials (MSMs) of the MCM-41 type were rapidly synthesized by microwave heating using silica fume as silica source and evaluated as adsorbents for the removal of Cu^2+^, Pb^2+^, and Cd^2+^ from aqueous solutions. The effects of microwave heating times on the pore structure of the resulting MSMs were investigated as well as the effects of different acids which were employed to adjust the solution pH during the synthesis. The obtained MCM-41 samples were characterized by nitrogen adsorption–desorption analyses, X-ray powder diffraction, and transmission electron microscopy. The results indicated that microwave heating method can significantly reduce the synthesis time of MCM-41 to 40 min. The MCM-41 prepared using citric acid (c-MCM-41(40)) possessed more ordered hexagonal mesostructure, higher pore volume, and pore diameter. We also explored the ability of c-MCM-41(40) for removing heavy metal ions (Cu^2+^, Pb^2+^, and Cd^2+^) from aqueous solution and evaluated the influence of pH on its adsorption capacity. In addition, the adsorption isotherms were fitted by Langmuir and Freundlich models, and the adsorption kinetics were assessed using pseudo-first-order and pseudo-second-order models. The intraparticle diffusion model was studied to understand the adsorption process and mechanism. The results confirmed that the as-synthesized adsorbent could efficiently remove the heavy metal ions from aqueous solution at pH range of 5–7. The adsorption isotherms obeyed the Langmuir model, and the maximum adsorption capacities of the adsorbent for Cu^2+^, Pb^2+^, and Cd^2+^ were 36.3, 58.5, and 32.3 mg/g, respectively. The kinetic data were well fitted to the pseudo-second-order model, and the results of intraparticle diffusion model showed complex chemical reaction might be involved during adsorption process.

**Electronic supplementary material:**

The online version of this article (doi:10.1186/s11671-017-2070-4) contains supplementary material, which is available to authorized users.

## Background

Heavy metal pollution has become a major environmental problem, threatening the environment and public health. Heavy metals can accumulate in the environment and cause heavy metal poisoning. They are not biodegradable and cannot be metabolized or decomposed. Moreover, they can easily enter the food chain and cause chronic toxic effects with gradual accumulation in living organisms. According to the World Health Organization drinking water guidelines, the acceptable concentration limits for Cu^2+^, Cd^2+^, and Pb^2+^ are 2, 0.003, and 0.01 mg/L, respectively [1]. Several methods have been applied for the effective removal of heavy metal ions such as ion exchange [2], nano-filtration [3], solvent extraction [4], chemical precipitation [5], adsorption [6, 7], etc. Among the available methods, adsorption technology is the most promising and frequently used technique due to its simplicity, high efficiency, and low cost [8]. Various kinds of adsorption materials have been used to remove heavy metal ions from aqueous solution, such as activated carbon [9] and clays [10]. However, these materials have low adsorption efficiency for heavy metal ions because of their low pore volume and poor pore structure.

Mesoporous materials have been widely used for the adsorption of heavy metal ions [11–14] due to their exceptionally large specific surface area, regular pore structure, and tunable pore sizes. Since the discovery of M41S silica in 1992 [15], MCM-41 has become the most popular type of M41S silica materials and one of the most commonly used mesoporous adsorption materials. MCM-41 is characterized by highly uniform pore channels, large pore size, and high surface area [16]. Synthesis of mesoporous MCM-41 materials attracts a lot of interest because of their potential applications in catalysis [17], ion exchange [18], biosensors [19], and drug delivery [20, 21]. Commercial reagents employed in the traditional preparation of MCM-41 are always expensive and toxic, such as tetraethylorthosilicate (TEOS) [22]. Moreover, other silica precursors, such as agricultural waste, have also been used to produce mesoporous silica materials (MCM-41). For instance, using rice husk ash for the synthesis of SBA-15, MCM-41, and MCM-48 has been reported previously [23–25]. The use of mesoporous materials from industrial solid waste, such as coal combustion waste fly ash, as a silica source has also attracted attention due to economic advantages [26, 27]. Silica fume, a very fine amorphous silica powder, is obtained as a by-product during metal production processes. Silica fume mainly contains amorphous SiO_2_ particles (greater than 85 wt%), and it has been used as an inexpensive silica source in the synthesis of microporous and mesoporous materials [28]. Thus, it further broadens the applications of silica fume which is generally used as an additive in cement and concrete [29], in bricks and ceramic tiles [30], as well as a filler in plastics and paints [31]. The use of silica fume as silica source also allows the green synthesis of MCM-41, since it does not require the use of any harmful reagents.

The conventional hydrothermal synthesis process of MCM-41 requires a long crystallization time and a high crystallization temperature. In 1996, thermally stable molecular sieve MCM-41 with hexagonal channels was synthesized in a temperature-controlled microwave oven from aged precursor gels within about 1 h by Wu et al. [32]. At present, microwave irradiation technique is widely applied to the synthesis of mesoporous molecular sieves [33–35]. Compared with the conventional hydrothermal synthesis method, microwave-assisted synthesis method employs microwave dielectric heating, uniform heating, or molecular selective heating. Thus, it offers the advantages of homogeneous heating and fast elevation of the temperature of synthesis system to crystallization temperature, resulting in more homogeneous nucleation, shorter crystallization times [36, 37], and products with uniform size [38, 39].

This work explores the preparation of mesoporous silica materials (of MCM-41 type) using a rapid microwave heating method and their application for Cu^2+^, Pb^2+^, and Cd^2+^ removal from aqueous solutions. The effects of different acids used for pH adjustment and different microwave heating times on the pore structure of MCM-41 were investigated. We also studied the influence of pH on adsorption capacity of the adsorbent. The metal adsorption isotherms were fitted using Langmuir and Freundlich adsorption isotherm models. In addition, kinetics and intraparticle diffusion model were also studied to understand the mechanism of the adsorption process.

## Methods

### Chemicals

Silica fume was used as the silica source and it was obtained from a local metallurgy-grade silicon factory. Its main chemical component (85 wt%) is SiO_2_. It was dried at 150 °C for 24 h and used after dissolution and purification; more details can be found in Additional file 1: Figure S1 and Table S1. Other reagents, such as sodium hydroxide (NaOH), cetyltrimethylammonium bromide (CTAB), hydrochloric acid (HCl), citric acid, oxalic acid, acetic acid, copper nitrate (Cu(NO_3_)_2_), lead nitrate (Pb(NO_3_)_2_), and cadmium nitrate (Cd(NO_3_)_2_) were purchased from Shanghai Chemical Reagent Company of China and used as received. All of the reagents were analytical grade.

### Preparation of MCM-41

The sodium silicate solution was conveniently obtained using dissolution method. Then, the MCM-41 was synthesized by the following procedure: 5 g of CTAB was dissolved in 100 mL of deionized water, and the aqueous solution was continuously stirred for 30 min at room temperature. Then, 50 mL of pretreatment filtrate was slowly introduced into the above CTAB solution under stirring, and then one of several different acids (HCl, citric acid, oxalic acid, or acetic acid) was added dropwise to adjust the pH value of the solution to an optimum range of 10.87–10.88. Subsequently, the solution was stirred for 30 min–1 h and then subjected to microwave heating (SANYO EM-208MS1, China) for different times to allow the hydrothermal reaction to occur. After crystallization of the product, it was then cooled to room temperature, filtered, and washed with deionized water. The obtained solid product was placed in an oven and dried at 110 °C for 12 h. Finally, the synthesized material was calcined at 550 °C for 5 h to remove the template molecules. MCM-41 samples synthesized via this route were designated as x-MCM-41(y), where “x” represents the acid used to adjust reaction solution pH and “y” is the microwave heating time.

### Characterization

The X-ray diffraction (XRD) patterns were recorded on a Rigaku D/MAX-3B diffractometer with Cu–Kα radiation with a voltage of 40 kV. Nitrogen (N_2_) adsorption–desorption isotherms were measured at 77.5 K by a NOVA 2200e surface area and pore size analyzer (Quantachrome Instruments). The specific surface area of the sample was calculated using BET (Brunauer–Emmett–Teller) method, and pore size distribution was determined using BJH (Berrett–Joyner–Halenda) model. The sample morphology was examined using a JEOL JEM-2100 transmission electron microscope (TEM).

### Adsorption Experiments

#### Effect of Initial pH

The effect of pH on Cu^2+^, Pb^2+^, and Cd^2+^ adsorption was investigated over the pH range from 3.0 ± 0.1 to 7.0 ± 0.1. In a typical procedure, the adsorption experiments of Cu^2+^, Pb^2+^, and Cd^2+^ were carried out in a series of conical flasks containing 0.1 g of c-MCM-41(40) and 100 mL of Cu^2+^, Pb^2+^, and Cd^2+^ solutions of 40 mg/L. The pH was adjusted to the desired value by HCl (0.1 M) or NaOH (0.1 M), and the pH values were measured by a pH meter (OHAUS Starter 3C, China). Then, the conical flasks were continuously stirred at 25 °C for 24 h. After reaching adsorption equilibrium, the suspended adsorbent was easily collected from the aqueous solution by centrifugation at a speed of 5000 rpm for 5 min. Then, the supernatant was filtered with a syringe filter of 0.22 μm and analyzed via an AAS spectrophotometer after appropriate dilution.

#### Adsorption Isotherm Experiment

Batch experiments were conducted to measure the adsorption isotherms of Cu^2+^, Pb^2+^, and Cd^2+^ over c-MCM-41(40). Typically, 0.1 g of c-MCM-41(40) and 100 mL of Cu(NO_3_)_2_, Pb(NO_3_)_2_ and Cd(NO_3_)_2_ solutions with different initial Cu^2+^, Pb^2+^, and Cd^2+^ concentrations (varying from 5 to 250 mg/L) were added to conical flasks and the adsorption was allowed to proceed at 25 °C under normal pH conditions with continuous stirring for 24 h. The equilibrium adsorption capacities of c-MCM-41(40) for Cu^2+^, Pb^2+^, and Cd^2+^ were calculated by the following equation:

1 $$ {q}_e={v}_0\left({C}_0- C\right)/ W $$

where *q*_*e*_ is the equilibrium adsorption capacity (mg/g), *C*_0_ and *C* are the initial and equilibrium concentrations (mg/L) of Cu^2+^, Pb^2+^, and Cd^2+^ solutions, respectively, *v*_0_ is the volume of the initial solution (L) used for sorption, and *W* is the weight of adsorbent (g).

#### Kinetic Adsorption Experiment

The equilibrium time was determined using 0.1 g of sorbent at 25 °C. The adsorption time was varied from 5 to 600 min. The adsorption kinetics were described by the pseudo-first-order model, pseudo-second-order model, and the intraparticle diffusion model. The pseudo-first-order model is generally expressed as follows:

2 $$ \ln \left({q}_e- q\right)= \ln {q}_e-{k}_1 t $$

where *q*_*e*_ and *q* are the sorption capacities (mg/g) at equilibrium and time *t* (min), respectively. *k*_*1*_ represents the rate constant (min^−1^) of the pseudo-first-order kinetic model. The pseudo-second-order model equation is given as:

3 $$ t/ q= t/{q}_e+1/{k}_2{q_e}^2 $$

where *k*_*2*_ is the rate constant (g/mg min) of the pseudo-second-order kinetic model.

The rate determining step of the sorption reaction was analyzed using the intraparticle diffusion model. The adsorption capacity was calculated according to the following equation:

4 $$ {q}_t={k}_i{t}^{1/2}+ C $$

where *q*_*t*_ is the adsorption capacity (mg/g) at different intervals, *C* is the intercept (mg/g), and *k*_*i*_ is the intraparticle diffusion rate constant (mg/g min^−0.5^) of the adsorption step.

## Results and Discussion

### Characterization of MCM-41

**Fig. 1:**
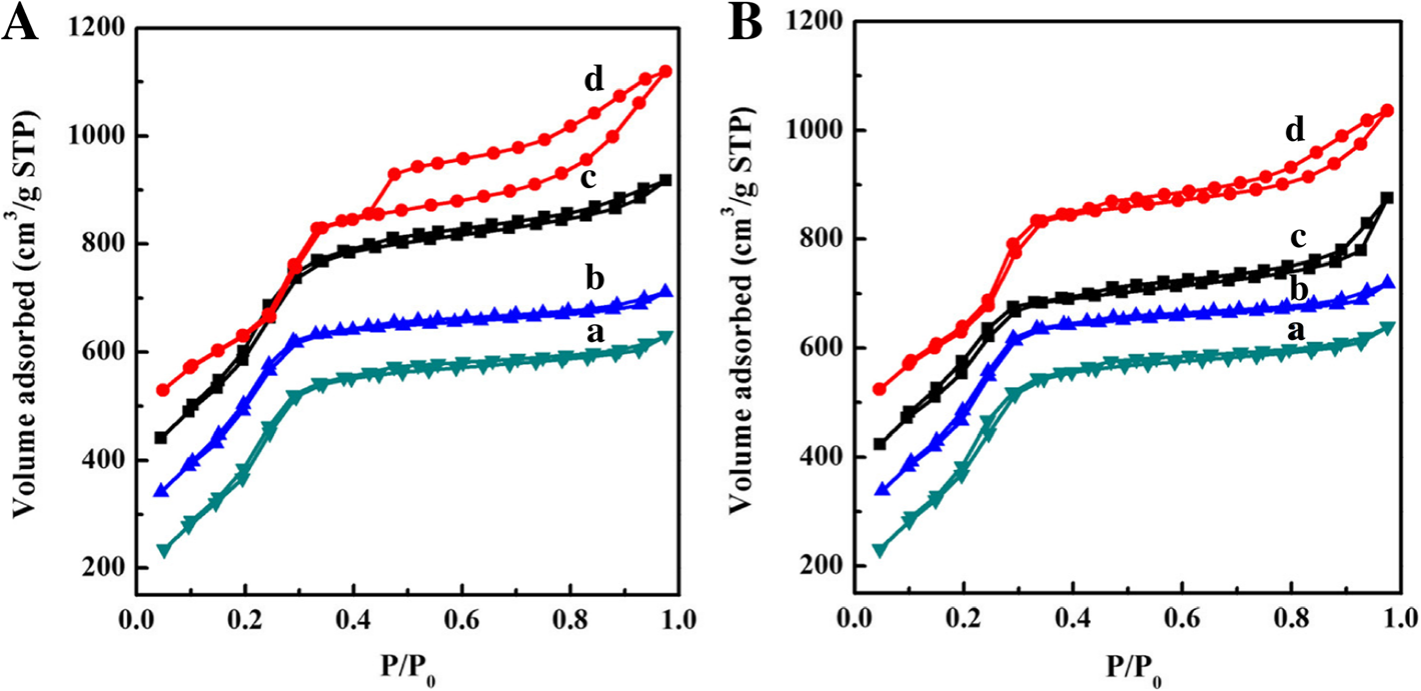
The N_2_ adsorption–desorption isotherms of the samples with different reaction times, **A** 40 min and **B** 60 min: *a* a-MCM-41, *b* o-MCM-41, *c* h-MCM-41, and *d* c-MCM-41. The isotherms shown in *b*, *c*, and *d* are offset by 100, 200, and 300 cm^3^/g, respectively

Nitrogen adsorption isotherms for the calcined MCM-41 samples prepared under different synthesis conditions are shown in Fig. 1 and Additional file 1: Figure S2. The isotherms of all samples exhibit the typical type IV mesopore sorption behavior of MCM-41 according to the IUPAC classification [40, 41]. All the isotherms clearly show a steep condensation step between the relative pressure range of 0.25 to 0.4 due to the condensation of nitrogen inside the primary mesopores. In a relatively high-pressure region of *p*/*p*_0_ > 0.4, gradual development of hysteresis loops can be observed, as the result of capillary condensation. Obviously, there is a broader hysteresis loop for the sample c-MCM-41(40) than others (Fig. 1a), suggesting that the pore shapes and sizes of c-MCM-41(40) are uniform. Furthermore, the surface areas (Table 1) of most samples decrease with increase in the crystallization time of the samples obtained by different acids because the high temperature causes the further condensation of Si–OH species. It is worth noting that the sample h-MCM-41 is more sensitive to the effect of synthesis time compared to the effect of organic acid (Fig. 1). When the heating time reached 60 min, the surface area of the sample h-MCM-41 quickly decreased to 1480.1 m^2^ g^−1^ (Table 1). From Table 1, it can be seen that the surface area of c-MCM-41 is lower than that of other samples, but the pore volume and pore diameter of c-MCM-41(40) are the highest among all samples. The likely reason for this phenomenon is that organic acids have slower ionization rates than HCl and also, the molecular size of citric acid is the largest among all the acids used in this study. It is also concluded that compared to room temperature synthesis (Additional file 1: Table S2), microwave heating synthesis method drastically reduces the reaction time to 40 min.

**Table 1:** Textural properties of the samples prepared with microwave heating

Sample	*S* (m^2^ g^−1^)	*V* (cm^3^ g^−1^)	*d* (nm)
h-MCM-41(40)	1633.6	0.29	3.3
c-MCM-41(40)	1253.5	0.65	3.6
o-MCM-41(40)	1652.1	0.16	3.6
a-MCM-41(40)	1601.5	0.18	3.6
h-MCM-41(60)	1480.1	0.36	3.6
c-MCM-41(60)	1296.2	0.39	3.6
o-MCM-41(60)	1583.3	0.16	3.6
a-MCM-41(60)	1564.3	0.19	3.6

**Fig. 2:**
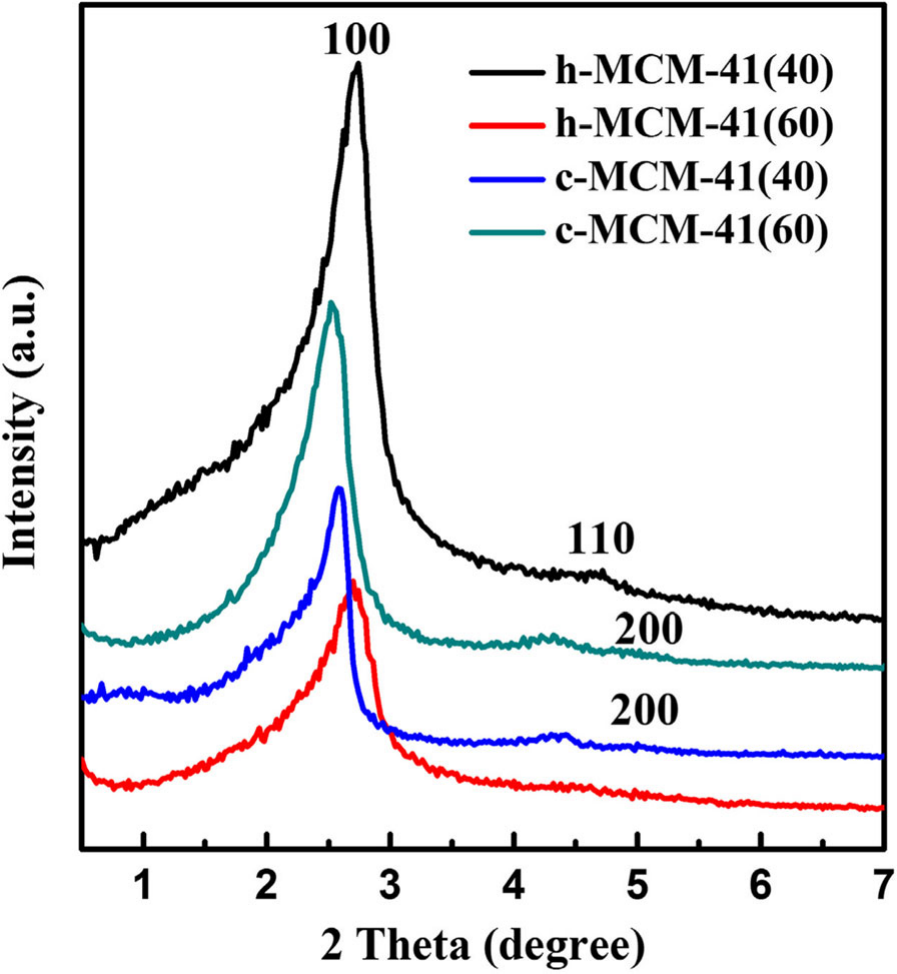
XRD patterns of the samples prepared at different reaction times with HCl and citric acid

Figure 2 shows the small-angle XRD patterns of samples synthesized with hydrochloric acid and citric acid under different microwave heating times. As shown in Fig. 2, all the samples have a well-defined diffraction peak around 2*θ* = 2.5 which can be indexed as (100) reflection and a diffraction peak (110) of much lower intensity around 2*θ* = 4.5, both of which are characteristic diffraction peaks of the typical MCM-41 mesoporous molecular sieve. Furthermore, both the two samples of c-MCM-41 have a weak diffraction peak related to the (200) plane suggesting that the sample has a highly ordered hexagonal pore structure. Additionally, the first diffraction peak (100) in h-MCM-41(40) exhibits the highest intensity among the four samples. When the time is increased to 60 min, the diffraction peak (100) becomes weak and the diffraction peak (110) becomes less distinct. On the other hand, there are no significant differences in the XRD patterns of the two samples of c-MCM-41. This phenomenon indicates that the long-range ordering of the sample synthesized with hydrochloric acid decreases as the crystallization time increases. On the other hand, samples synthesized with citric acid mostly retain their long-range ordering, which is in agreement with the nitrogen adsorption isotherms.

**Fig. 3:**
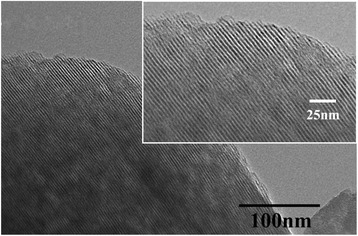
TEM image of c-MCM-41(40)(perpendicular to the channel)

Figure 3 and Additional file 1: Figure S3 present the TEM images of the sample c-MCM-41(40). It can be seen the regular stripes in the vertical direction of the channel, which is a long-range structure of the one-dimensional channel of MCM-41 (Fig. 3); along the channel, it can be seen the regular hexagonal array of the cross section of mesoporous channels (Additional file 1: Figure S3). The pore size is measured to be 2.68 nm. It is also in agreement with the nitrogen adsorption isotherms. TEM observation further indicates that the samples synthesized with citric acid possess better mesoporous structure than those prepared using hydrochloric acid (Additional file 1: Figure S4).

### Effect of Solution pH

**Fig. 4:**
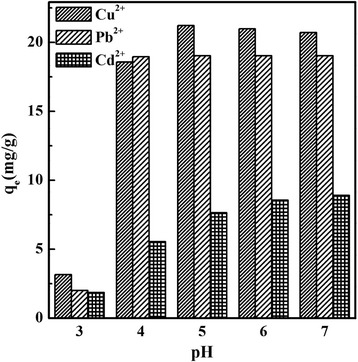
The effect of pH on Cu^2+^, Pb^2+^, and Cd^2+^ removal using c-MCM-41(40)

**Fig. 5:**
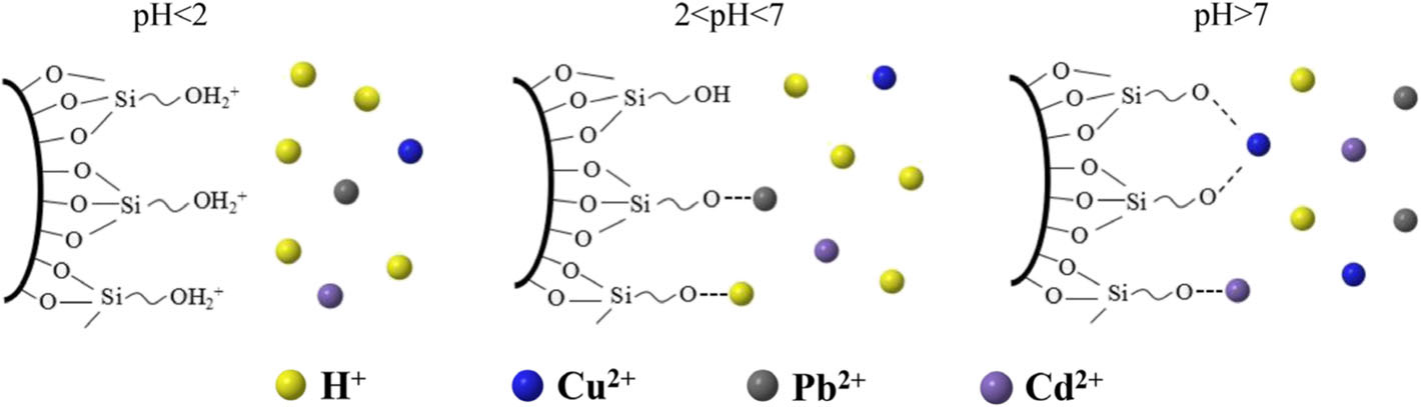
Schematic diagrams of heavy metal adsorption mechanisms for mesoporous silica under different pH value conditions

The pH of the solution plays an important role in determining the adsorption ability of MCM-41 for heavy metals. The effect of pH on the adsorption capacity of c-MCM-41(40) for Cu^2+^, Pb^2+^, and Cd^2+^ was investigated, and the corresponding results are presented in Fig. 4. As shown in Fig. 4, the adsorption capacities for Cu^2+^, Pb^2+^, and Cd^2+^ increase with the increase in pH value. When pH is less than 4.0, the adsorption capacities for Cu^2+^, Pb^2+^, and Cd^2+^ are low, while the maximum adsorption capacities for Cu^2+^ and Pb^2+^ were obtained at pH 5.0 reaching 21 and 19.02 mg/g, respectively. The maximum adsorption capacity for Cd^2+^ was 8.9 mg/g in the pH region of 6.0–7.0. This influence of pH on the adsorption can be explained by the isoelectric point of silica material which is about pH = 2, determined from its Zeta potential. When the pH value is below 2, the silica surface is positively charged, so there is electrostatic repulsion between the adsorbent and Cu^2+^, Pb^2+^, and Cd^2+^. The lower the pH value, the higher the repulsion. However, when the pH value is more than 2, the silica surface is negatively charged; thus, the inner channels and particle surface of MCM-41 are negatively charged, which leads to the adsorption and removal of Cu^2+^, Pb^2+^, and Cd^2+^ through electrostatic attraction. Positively charged ions such as H^+^ and the heavy metal ions Cu^2+^, Pb^2+^, and Cd^2+^ in the solution are strongly attracted to the negatively charged groups in the channels and surface of MCM-41 when the pH is acidic, causing competitive adsorption between H^+^ and heavy metal ions Cu^2+^, Pb^2+^, and Cd^2+^. The adsorption active sites on the MCM-41 surface are occupied by H^+^ reducing the adsorption of Cu^2+^, Pb^2+^, and Cd^2+^. With further increase in the solution pH, the H^+^ will combine with silanol groups on the MCM-41 surface and dissociate, which will increase the number of active sites for adsorption as well as the activity of hydroxyl groups on the MCM-41 surface. This in turn enhances the overall negative charge of MCM-41 and also improves the hydrolysis ability of the heavy metal ions in solution. These effects lead to the increase in the removal percentage and adsorption capacity of heavy metal ions. As for Cu^2+^, while pH < 5.77, the decrease of Cu^2+^ in the solution is due to the adsorption of adsorbent; but while pH > 5.77, the reduction of Cu^2+^ concentration is due to both effects of adsorption and precipitation, and the precipitation is dominant. More details you can see in Additional file 1 [Formula (S1)]. Figure 5 shows the heavy metal adsorption on mesoporous silica under different pH value conditions.

### Adsorption Isotherms

**Fig. 6:**
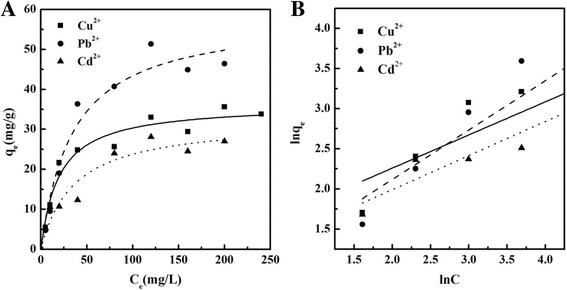
Adsorption isotherms of Cu^2+^, Pb^2+^, and Cd^2+^ on c-MCM-41(40). **A** Langmuir. **B** Freundlich

Two isotherm equations (Langmuir and Freundlich) have been tested in this study. The nonlinear and linear equations were used to fit the Cu^2+^, Pb^2+^, and Cd^2+^ adsorption process on c-MCM-41(40), as shown in Fig. 6. The model parameters are listed in Additional file 1: Table S2. The Langmuir isotherm assumes a homogeneous adsorption process while the Freundlich adsorption isotherm is an empirical equation employed to describe heterogeneous systems. Results show that the Langmuir model is better than the Freundlich model in simulating the adsorption experiments. This suggests that the adsorption process is a homogeneous process. The maximum adsorption capacities for Cu^2+^, Pb^2+^, and Cd^2+^ were 36.3, 58.5, and 32.3 mg/g, respectively, which are higher than or at least comparable with the already published data (Table 2).

**Table 2:** Comparison of Cu^2+^, Pb^2+^, and Cd^2+^ sorption capacities with other sorbents

Adsorbent	Adsorption capacity			References
	Cu^2+^ (mg/g)	Pb^2+^ (mg/g)	Cd^2+^ (mg/g)	
Coffee grounds	–	–	15.65	J. Hazard. Mater., 184 (2010) 126–134
Red mud	19.72	–	10.57	Water Research, 32(4) (1998) 1314–1322
Soybean straw	5.40	6.84	2.02	Desalination, 229 (2008) 170–180
Corn stalk	3.75	6.01	5.17	
Dried sludge	–	–	6.6	Appl. Water Sci., 3 (2013) 321
Nano-γ-Al_2_O_3_	–	6	1.1	J. Environ. Sci. Technol., 12 (2015) 2003–2014
Activated poplar sawdust	9.2	–	–	J. Hazard. Mater., 137 (2006) 909–914
Spent-activated clay	10.9	–	–	Sep. Purif. Technol., 54 (2007) 187–197
Wheat straw	4.96	9.74	5.17	Proc. 1999 Conference on Hazardous Waste Research, St. Louis, 1999, pp. 121–130
Oat straw	5.15	18.86	4.72	

### Kinetic Study

The kinetics of Cu^2+^, Pb^2+^, and Cd^2+^ adsorption on c-MCM-41(40) are shown in Additional file 1: Figure S5. The adsorption of all heavy metals rapidly occurred within 50 min due to the large number of available sites at the initial stage. With the increase in adsorption time, there was a gradual decrease in the concentration of heavy metals in solution as well as the available active sites on adsorbent, due to the accumulation of metal ions on c-MCM-41(40), leading to the decrease in adsorption rate at the later stage.

Adsorption rate has been analyzed by using two common semi-empirical kinetic models which are based on adsorption equilibrium capacity: the pseudo-first-order and pseudo-second-order equations. Table 3 summarizes the corresponding parameters, and the correlation fitting curves are shown in Additional file 1: Figure S6. Comparing the *R* ^2^ values derived from both models, it is found that the pseudo-second-order kinetic model fits better with the kinetic data of c-MCM-41(40) than the pseudo-first-order kinetic model. In addition, the calculated equilibrium adsorption capacities (*q*_e,cal_) from the pseudo-second-order adsorption kinetics are very close to the experimental data (*q*_e,exp_).

**Table 3:** Kinetic parameters and correlation coefficients of the two kinetic equations on c-MCM-41(40)

Metal ions	*q*_e.exp_ (mg/g)	First-order kinetic model			Second-order kinetic model		
		*a*_e,cal_ (mg/g)	*K*_1_	*R* ^2^	*q*_e,cal_ (mg/g)	*K*_2_	*R* ^2^
Cu^2+^	11.57	2	0.2758	0.6427	11.61	0.5733	0.9975
Pb^2+^	18.7	5.74	0.2401	0.8839	19.4	0.1467	0.9998
Cd^2+^	9.25	1.83	0.199	0.6231	9.44	0.291	0.9909

### Intraparticle Diffusion Model

**Fig. 7:**
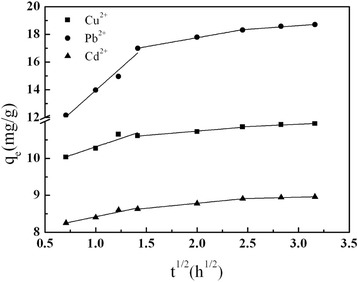
Plot of interparticle diffusion model for adsorption of Cu^2+^, Pb^2+^, and Cd^2+^ onto the c-MCM-41(40)

The rate-limiting step of the adsorption was analyzed using an intraparticle diffusion model. As shown by the model, if the rate-limiting step of the adsorption process is intraparticle diffusion, the plots of *q*_*t*_ versus *t* ^1/2^ will be a straight line, which will pass through the point of origin. Figure 7 shows that the plots of *q*_e_ versus *t* ^1/2^ of Cu^2+^, Pb^2+^, and Cd^2+^ displayed a stepwise-linear pattern with three slopes. The values of *k*_*i*_ and *C* calculated from the slope and intercept of *q*_e_ versus *t* ^1/2^ plots are listed in Additional file 1: Table S3. The results showed that three steps with different limiting processes were involved in adsorption process. These steps included the instantaneous or external surface adsorption at the early stage, the gradual adsorption stage where intraparticle diffusion into the mesopores and micropores was the rate-limiting step, and the final stage where intraparticle diffusion slowed down because of the relatively low residual heavy metal ions concentration in the solution. The second stage did not pass through the origin, which suggested that the intraparticle diffusion was not the only rate-limiting step, and complex reaction might also be involved.

## Conclusions

In this study, an ordered mesoporous silica material MCM-41 has been conveniently synthesized using silica fume as the silica source under microwave heating. Microwave heating synthesis method significantly reduced the reaction time to 40 min. The sample c-MCM-41(40) prepared with citric acid under microwave heating for 40 min possesses the highest pore volume and pore diameter among all the samples, as well as well-defined crystallinity and regular hexagonal array of mesoporous channels. It also shows good performance for removing Cu^2+^, Pb^2+^, and Cd^2+^ in the pH region of 5.0–7.0. The adsorption data of Cu^2+^, Pb^2+^, and Cd^2+^ showed good fitting with the Langmuir isotherm, suggesting that the adsorption process is a homogeneous process. The maximum adsorption capacities of c-MCM-41(40) for Cu^2+^, Pb^2+^, and Cd^2+^ were 36.3, 58.5, and 32.3 mg/g, respectively. Kinetic data of c-MCM-41(40) were found to fit better with pseudo-second-order kinetic model than pseudo-first-order kinetic model, and the calculated equilibrium adsorption capacities (*q*_e,cal_) from the pseudo-second-order adsorption kinetics were very close to the experimental data (*q*_e.exp_). In addition, the results of intraparticle diffusion model indicate that the intraparticle diffusion is not the only rate-limiting step, and complex chemical reactions or redox reactions might also be involved.

## Additional file

Additional file 1: Figure S1. schematic diagram of the dissolution of silica fume. **Table S1.** Purification rate of silica fume. **Figure S2.** the N_2_ adsorption–desorption isotherm of the sample prepared with HCl at room temperature within 24 h. **Table S2.** Textural properties of the sample prepared with HCl at room temperature within 24 h. **Figure S3.** TEM image of c-MCM-41(40) (along the channel). **Figure S4.** TEM image of h-MCM-41(40). **Table S3.** Fitting parameters of Langmuir and Freundlich isotherms for the adsorption of Cu^2+^, Pb^2+^, and Cd^2+^ on c-MCM-41(40). **Figure S5.** The kinetics of Cu^2+^, Pb^2+^, and Cd^2+^ adsorption on c-MCM-41(40). **Figure S6.** Plot of kinetic model for the adsorption of Cu^2+^, Pb^2+^, and Cd^2+^, (A) pseudo-first-order kinetic model (B) pseudo-second-order kinetic model. (DOCX 1066 kb)
