# Supplementary material for: Investigating the Heavy Metal Adsorption of Mesoporous Silica Materials Prepared by Microwave Synthesis
Source: Nanoscale Res Lett. 2017 May 4;12:323. doi: 10.1186/s11671-017-2070-4 (PMC5418173; doi:10.1186/s11671-017-2070-4)
Supplement: Additional file 1: Figure S1. — schematic diagram of the dissolution of silica fume. Table S1. Purification rate of silica fume. Figure S2. the N2 adsorption–desorption isotherm of the sample prepared with HCl at room temperature within 24 h. Table S2. Textural properties of the sample prepared with HCl at room temperature within 24 h. Figure S3. TEM image of c-MCM-41(40) (along the channel). Figure S4. TEM image of h-MCM-41(40). Table S3. Fitting parameters of Langmuir and Freundlich isotherms for the adsorption of Cu2+, Pb2+, and Cd2+ on c-MCM-41(40). Figure S5. The kinetics of Cu2+, Pb2+, and Cd2+ adsorption on c-MCM-41(40). Figure S6. Plot of kinetic model for the adsorption of Cu2+, Pb2+, and Cd2+, (A) pseudo-first-order kinetic model (B) pseudo-second-order kinetic model. (DOCX 1066 kb) [file 11671_2017_2070_MOESM1_ESM.docx]

***Supporting Information***

Title:

**Investigating the heavy metal adsorption of mesoporous silica materials prepared by microwave synthesis**

Wenjie Zhu^1,2,4*^, Jingxuan Wang^1^, Di Wu^1^, Xitong Li^1^, Yongming Luo^1^, Caiyun Han^1^, Wenhui Ma^2,3,4^, Sufang He^5^

^1^ Faculty of Environmental Science and Engineering, Kunming University of Science and Technology, Kunming 650500, China

^2^ Faculty of Metallurgical and Energy Engineering, Kunming University of Science and Technology, Kunming 650093, China

^3^ State Key Laboratory of Complex Nonferrous Metal Resources Cleaning Utilization in Yunnan Province/The National Engineering Laboratory for Vacuum Metallurgy, Kunming University of Science and Technology, Kunming 650093, China

^4^ Key Laboratory of Non-Ferrous Metals Vacuum Metallurgy of Yunnan Province/Engineering Research Center for Silicon Metallurgy and Silicon Materials of Yunnan Provincial Universities, Kunming 650093, China

^5^ Research Center for Analysis and Measurement, Kunming University of Science and Technology, Kunming 650093, China

* Corresponding author

Tel: +86-871-65103845; Fax: +86-871-65103845;

E-mail: zhuwenjie17@163.com

Purify silica source

Certain quality of silica fume and NaOH were mixed into 80 mL of deionized water at a molar ratio of 1 : 2.2 and then the mixture was stirred in water bath at 90°C for 3 h, then the mixture was filtered to obtain sodium silicate solution. Fig. S1 is schematic diagram of the dissolution of silica fume.

4g of silica fume and various quality of NaOH were used to extract silica from silica fume and the corresponding purification rate were list in Table S1. In order to make full use of silica fume and save the cost of material synthesis, we finally determine the silica fume/NaOH quality ratio is 4:3 for the later experiment.


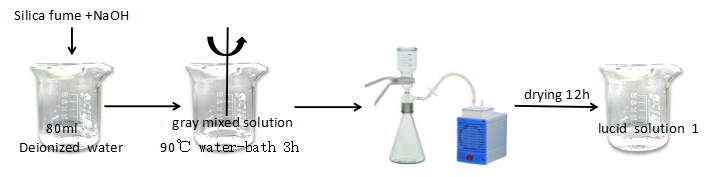


Fig. S1 schematic diagram of the dissolution of silica fume

Table S1 Purification rate of silica fume

| quality ratio of Si/NaOH | purification rate of silica fume |
| --- | --- |
| 4:3 | 93.0% |
| 4:4 | 94.4% |
| 4:5 | 94.7% |
| 4:6 | 92.8% |
| 4:8 | 95.2% |


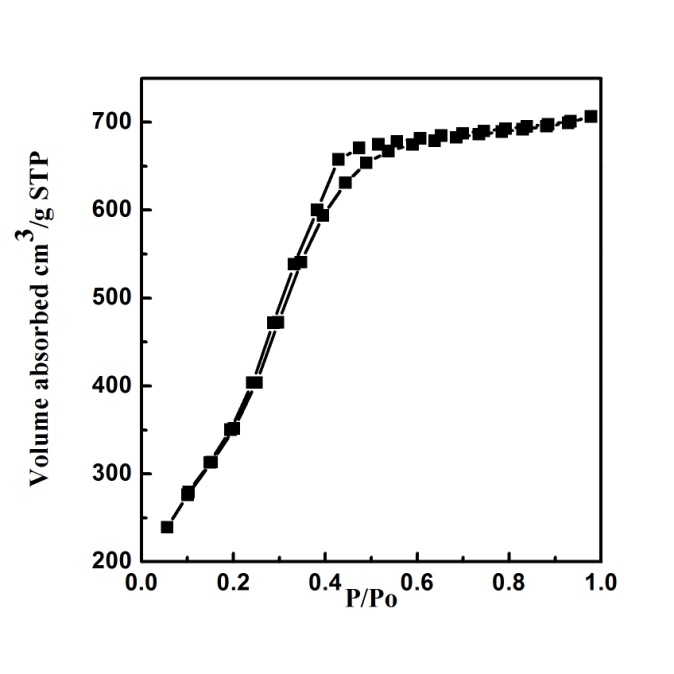


Fig. S2 the N_2_ adsorption-desorption isotherm of the sample prepared with HCl at room temperature within 24 h.

Table S2 Textural properties of the sampl prepared with HCl at room temperature within 24 h.

| Sample | *S* (m^2^g^-1^) | *V* (cm^3^g^-1^) | *d* (nm) |
| --- | --- | --- | --- |
| h-MCM-41(24h) | 1404.492 | 0.322 | 3.264 |


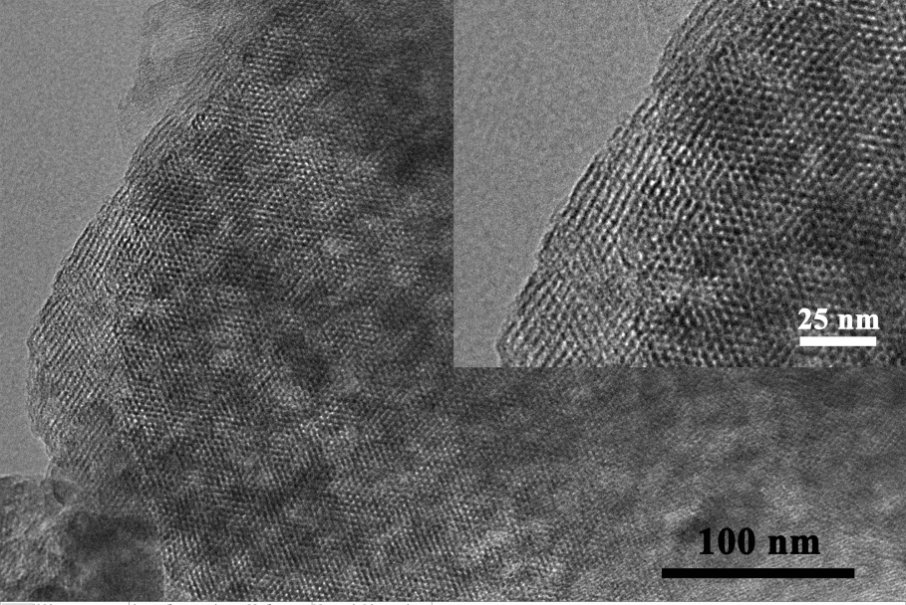


Fig. S3 TEM image of c-MCM-41(40)(along the channel).





Fig. S4 TEM image of h-MCM-41(40).

Calculation of pH value

The initial concentration of Cu^2+^, Pb^2+^ and Cd^2+^ solutions is 40 mg/L, the pH range is from 3.0 ± 0.1 to 7.0 ± 0.1 in this paper. Different metal ions will produce precipitation under the different pH value. the pH value at the beginning of precipitation have been calculated by Formula (S1). In the formula (S1), K_sp_^θ^ is the solubility product constant of metal hydroxide, K_w_^θ^is the ion product of water, [M^n+^] is metal ion concentration.

The detailed calculation process is as follows:

$\left[\text{Cu}^{2+}\right]=\frac{40\times10^{-3}}{63.546}=6.3\times10^{-4}$ $K_{sp}^{\theta}[\text{Cu(OH)}_{2}]=10^{-19.66}$

$\left[\text{Pd}^{2+}\right]=\frac{40\times10^{-3}}{207.2}=1.93\times10^{-4}$ $K_{sp}^{\theta}[\text{Pd(OH)}_{2}]=10^{-14.84}$

$\left[\text{Cd}^{2+}\right]=\frac{40\times10^{-3}}{112.4}=3.56\times10^{-4}$ $K_{sp}^{\theta}[\text{Cd(OH)}_{2}]=10^{-14.14}$

Precipitation principle of metal hydroxides: $\text{M(OH)}_{\text{n}}\Leftrightarrow\text{M}^{\text{n}+}+\text{nOH}^{-}$

The solubility product of the corresponding hydroxide: $K_{sp}^{\theta}=[\text{M}^{\text{n}+}][\text{OH}^{-}]^{n}$

While, $K_{W}^{\theta}=[\text{H}^{+}][\text{OH}^{-}]$

So the pH value of the metal hydroxide which starts to precipitate can be obtained:

$\text{pH}=\frac{1}{n}*\lg K_{sp}^{\theta}-\lg K_{W}^{\theta}-\frac{1}{n}*\lg[\text{M}^{\text{n}+}]$ (S1)

For instance, $\text{Cu(OH)}_{2}\Leftrightarrow\text{Cu}^{2+}+\text{2OH}^{-}$ $K_{sp}^{\theta}=\text{[Cu}^{2+}\text{][OH}^{-}\text{]}^{2}$

$$\text{pH}=\frac{1}{2}*\lg(10^{-19.66})-\lg(1.0\times10^{-14})-\frac{1}{2}*\lg(6.3\times10^{-4})=5.77$$

Similarly, $\text{Pb(OH)}_{2}:\text{pH}=\frac{1}{2}*\lg(10^{-14.84})-\lg(1.0\times10^{-14})-\frac{1}{2}*\lg(1.93\times10^{-4})=8.44$

$$\text{Cd(OH)}_{2}\text{:pH}=\frac{1}{2}*\text{lg(10}^{-14.14})-\lg(1.0\times10^{-14})-\frac{1}{2}*\lg(3.56\times10^{-4})=8.65$$

Hence, Pb^2+^ and Cd^2+^ begin to precipitate while the pH values are 8.44 and 8.65, both of them do not precipitate between the pH range in this investagation. As for Cu^2+^, while pH<5.77, the decrease of Cu^2+^ in the solution is due to the adsorption of adsorbent; but while pH>5.77, the reduction of Cu^2+^ concentration is due to both effect of adsorption and precipitation, and the precipitation is dominant.

Table S3 Fitting parameters of Langmuir and Freundlich isotherms for the adsorption of Cu^2+^, Pb^2+^ and Cd^2+^ on c-MCM-41(40).

| Metal ions | Langmuir | | |  | Freundlich | | |
| --- | --- | --- | --- | --- | --- | --- | --- |
|  | $Q_{m}$/*mg·g*^-1^ | $K_{L}$(L/mmol) | R^2^ |  | K | 1/n | R^2^ |
| Cu^2+^ | 36.3 | 3.2 | 0.9625 |  | 4.15 | 0.4131 | 0.835 |
| Pb^2+^ | 58.5 | 6.0 | 0.96 |  | 2.43 | 0.6153 | 0.89 |
| Cd^2+^ | 32.3 | 3.14 | 0.886 |  | 3.13 | 0.425 | 0.909 |


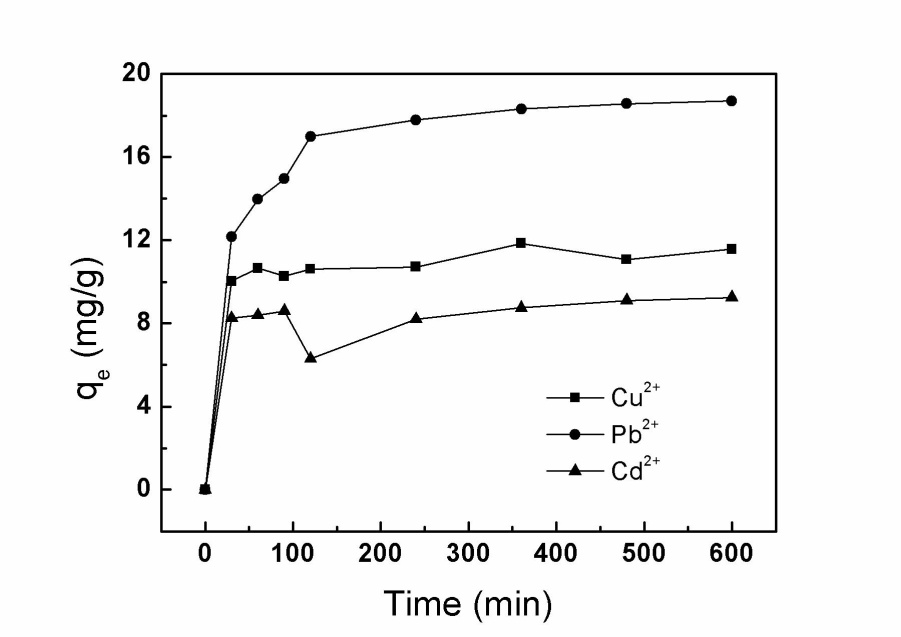


Fig. S5 The kinetics of Cu^2+^, Pb^2+^ and Cd^2+^ adsorption on c-MCM-41(40).


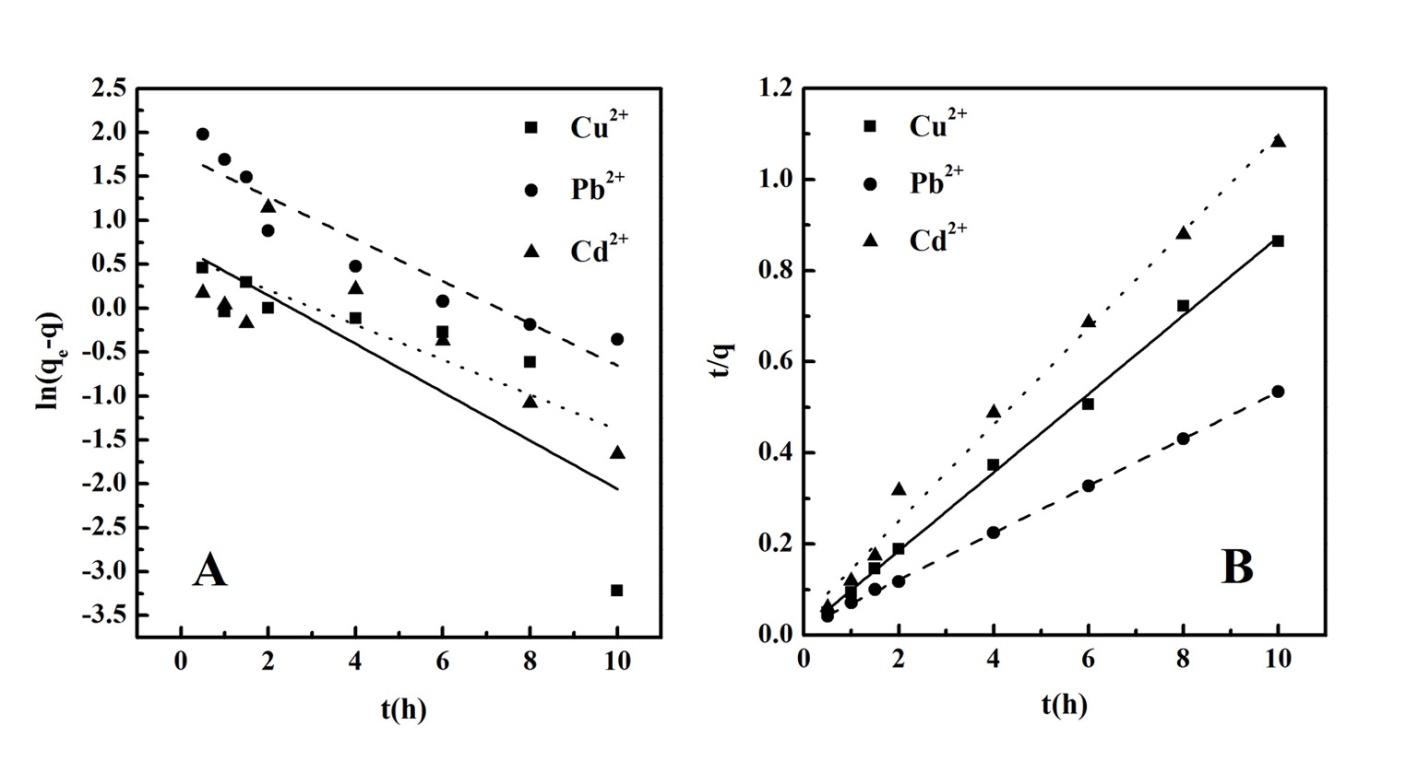


Fig. S6 Plot of kinetic model for the adsorption of Cu^2+^, Pb^2+^ and Cd^2+^, (A) pseudo-first-order kinetic model (B) pseudo-second-order kinetic model.

Table S4. The fitted parameters of intraparticle diffusion model for the adsorption of Cu^2+^, Pb^2+^ and Cd^2+^ on c-MCM-41(40).

| Metal ions | *k_i,_*_1_ mg/g min^-0.5^ | C_1_ | R^2^ | *k_i,_*_2_ mg/g min^-0.5^ | C_2_ | R^2^ | *k_i,_*_3_ mg/g min^-0.5^ | C_3_ | R^2^ |
| --- | --- | --- | --- | --- | --- | --- | --- | --- | --- |
| Cu^2+^ | 0.918 | 9.39 | 0.847 | 0.23 | 10.272 | 0.969 | 0.127 | 10.542 | 0.953 |
| Pb^2+^ | 6.524 | 7.431 | 0.958 | 1.29 | 15.181 | 0.997 | 0.536 | 17.022 | 0.959 |
| Cd^2+^ | 0.575 | 7.844 | 0.941 | 0.269 | 8.246 | 0.998 | 0.07 | 8.739 | 0.988 |
